# Integrin β3 and CD44 levels determine the effects of the OPN-a splicing variant on lung cancer cell growth

**DOI:** 10.18632/oncotarget.10865

**Published:** 2016-07-27

**Authors:** Shih-Jung Sun, Chun-Chi Wu, Gwo-Tarng Sheu, Hui-Yi Chang, Mei-Yu Chen, Yu-Ying Lin, Cheng-Yen Chuang, Shih-Lan Hsu, Jinghua Tsai Chang

**Affiliations:** ^1^ Institute of Medicine, Chung Shan Medical University, Taichung, Taiwan, ROC; ^2^ Institute of Medical and Molecular Toxicology, Chung Shan Medical University, Taichung, Taiwan, ROC; ^3^ Division of Thoracic Surgery, Taichung Veterans General Hospital, Taichung, Taiwan, ROC; ^4^ Department of Education & Research, Taichung Veterans General Hospital, Taichung, Taiwan, ROC; ^5^ Department of Chest Medicine, Chung Shan Medical University Hospital, Taichung, Taiwan, ROC

**Keywords:** OPN-a, Integrin beta3, CD44, NF-kB

## Abstract

Osteopontin (OPN), a phosphorylated glycoprotein, is frequently overexpressed in cancer. Among the three OPN isoforms, OPN-a is the most highly expressed in lung cancer cell lines and lung tumors. Overexpression of OPN-a greatly reduced CL1-5 lung adenocarcinoma cell growth, but had no effect on growth in A549 lung adenocarcinoma cells. Examination of the expression of integrins and CD44, which are possible OPN-a receptors, revealed that differences in integrin β3 levels might explain this discrepancy between CL1-5 and A549 cells. When integrin β3 was ectopically expressed in A549 cells, OPN-a inhibited their growth, whereas OPN-a increased cell growth following integrin β3 knockdown in CL1-5 cells. This OPN-a-induced increase in growth appeared to result from activation of the CD44/NFκB pathway. Our results demonstrated that OPN-a inhibits growth of cells with high integrin β3 levels and increases growth via activation of the CD44/NFκB pathway in cells with low integrin β3 levels. Thus, OPN-a, integrin β3, and CD44 interact to affect lung cancer cell growth, and this study may aid in the development of cancer treatment strategies involving these molecules.

## INTRODUCTION

Osteopontin (OPN) is a multifunctional phosphorylated glycoprotein and an inducible marker secreted by transformed, malignant epithelial cells [1]. It is expressed in a wide range of cells and regulates many biological functions, including immune responses, tissue remodeling, and vascularization, through its effects on two types of cell adhesion molecules: integrins [2, 3] and CD44 [4, 5]. OPN is also involved in tumor-associated proliferation [6, 7], survival [8], adhesion [9, 10], migration [11, 12], invasion, and angiogenesis [13]. Increased expression of OPN is associated with reduced progression and metastasis in lung [14, 15], breast [11, 16], colon [17], liver [18], stomach [12], and prostate [19] cancer.

**Figure 1:**
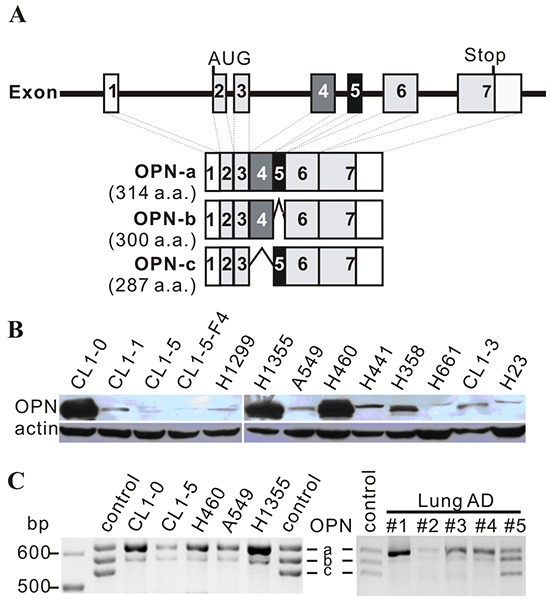
OPN expression in cell lines **A.** Schematic diagram of OPN isoforms. OPN-a is the full-length isoform (314 a.a.), OPN-b lacks exon 5 (300 a.a.), and OPN-c is missing exon 4 (287 a.a.). **B.** Twenty μg of total protein were subjected to Western blot analysis. OPN expression was low in several highly-invasive cell lines, including CL1-5, CL1-5-F4, H1299, and A549. **C.** Expression of OPN isoforms in various lung cancer cell lines and lung adenocarcinoma (AD) specimens. Control bands were mixed isoforms amplified from OPN-a, OPN-b, and OPN-c constructs via PCR.

Several isoforms of OPN transcripts have been identified, including OPN-a (full-length form), OPN-b (lacking exon 5), and OPN-c (lacking exon 4) (Figure 1A) [20]. Numerous studies addressing the roles of these isoforms in tumorigenesis yielded conflicting results. In breast cancer, OPN-c is more effective in enhancing anchorage-independent growth than OPN-a [21]. In hepatocellular carcinoma cell lines, OPN-a and -b, but not OPN-c, induce cell migration in SK-Hep1 cells with robust migratory capacity. Conversely, OPN-c, but not OPN-a, represses migration in Hep3B cells. OPN-c also promotes the formation of adhesive foci in Hep3B cells [22]. In PC-3 prostate cancer cells, OPN-b and OPN-c overexpression promote tumorigenesis [23]. In non-small cell lung cancer (NSCLC) cell lines, OPN-a and OPN-b enhance, while OPN-c suppresses, cell invasion. Moreover, while OPN-a enhances and OPN-c suppresses colony formation, OPN-b has inconsistent effects [24].

We have observed that 65% of NSCLC specimens express high levels of OPN protein [25]. The predominant form of OPN expressed in lung cancer cell lines and lung tumors is OPN-a. In the absence of integrin β3 (ITGβ3), OPN-a enhances cell growth through the CD44/NFκB pathway. When OPN-a levels are low, ITGβ3 is essential for CL1-5 lung cancer cell growth. However, OPN-a inhibits lung cancer cell growth in the presence of high levels of ITGβ3. The aim of this study was to investigate the effects of ITGβ3 and CD44, two important growth-enhancing molecules in lung cancer cells, on the regulatory role of OPN-a in lung cancer cell growth.

## RESULTS

### OPN splicing affects lung cancer cell growth

Overexpression of OPN has been implicated in tumor growth and invasion in various cancers, including lung cancers. However, a number of highly-invasive lung cancer cell lines, such as A549, H1299, and CL1-5, expressed low levels of OPN protein (Figure 1B). Among the three OPN isoforms, OPN-a was the most highly-expressed isoform in lung cancer cell lines and tumors (Figure 1C).

**Figure 2:**
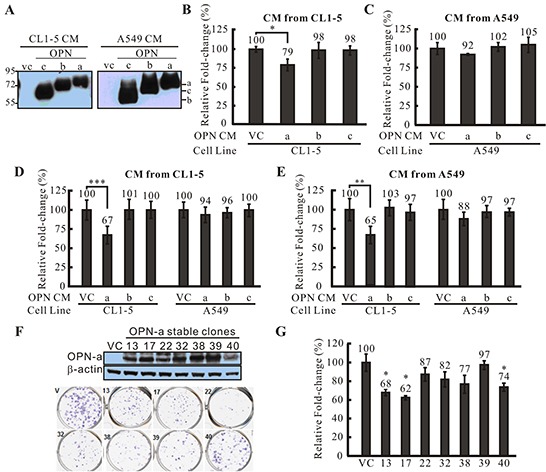
The effect of OPN-a on cell growth **A.** Overexpressed OPN splicing variants were secreted into medium (CM) at similar levels. VC represents cells transfected with empty vector. **B.** CM from CL1-5 cells transiently transfected with various OPN splicing variants or VC was used to treat other CL1-5 cells. CM/OPN-a strongly inhibited CL1-5 cell growth, while CM containing OPN-b or OPN-c had little or no effect. Fold-changes in cell numbers were determined by dividing cell numbers at 24 h by those at 0 h. Relative fold-change in cell number was calculated by dividing each fold-change in CM/OPN treatment cells by the fold-change in CM/VC cells. *, *p* < 0.05. **C.** CM collected from A549 cells transiently transfected with various OPN splicing variants or VC was used to treat other A549 cells. In contrast to CL1-5 cells, CM containing OPN-a, OPN-b, or OPN-c did not inhibit A549 cell growth. **D.** CM containing various OPN splicing variants was collected from CL1-5 cells and used to treat CL1-5 and A549 cells. CM/OPN-a from CL1-5 cells inhibited growth of CL1-5, but not A549, cells. ***, *p* < 0.001. **E.** CM containing various OPN splicing variants was collected from A549 cells and used to treat CL1-5 and A549 cells. CM/OPN-a from A549 cells inhibited growth of CL1-5, but not A549, cells. **, *p* < 0.01. **F.** Expression of exogenous OPN-a in CL1-5 stable clones. All CL1-5/OPN-a stable clones showed low focus formation capacity compared to vector controls (VC). **G.** CL1-5 stable clones overexpressing OPN-a showed slower growth rates compared to VC. *, *p* < 0.05.

OPN-a, OPN-b, and OPN-c splicing variants were cloned and transiently transfected into CL1-5 and A549 cells. These ectopically expressed OPN proteins were also secreted into culture medium (Figure 2A). To determine the effects of the different OPN splicing variants on cell growth, we treated CL1-5 cells with conditioned medium (CM) from other CL1-5 cells transiently transfected with different OPN isoforms. Surprisingly, CL1-5 cells treated with CM/OPN-a had a slower growth rate than cells treated with medium collected from empty vector controls (VC). Treatment with CM containing OPN-b or OPN-c had little or no effect on CL1-5 cell growth (Figure 2B). We also conducted the same experiment in A549 cells. Unexpectedly, A549 CM/OPN-a did not inhibit growth in A549 cells (Figure 2C). It is possible that overexpression of OPN-a induces the secretion of different molecules in CL1-5 and A549 cells, causing different cell growth responses. Alternatively, this discrepancy in the effect of OPN-a on growth in CL1-5 and A549 cells may be explained by differences in receptor or down-stream effector expression levels. Thus, CL1-5 and A549 cells were treated with CM collected from CL1-5 (Figure 2D) or A549 (Figure 2E) cells expressing different OPN splicing variants. CM/OPN-a from either CL1-5 or A549 cells suppressed CL1-5, but not A549, cell growth.

Next, we screened CL1-5 cells stably expressing OPN-a with G418 (Figure 2F) and performed focus formation assays and cell growth analysis on the OPN-stable clones. In agreement with CM treatment results, CL1-5 cells stably expressing OPN-a generated fewer and smaller foci compared to VC cells (Figure 2F). In addition, growth rates were lower in CL1-5 cells ectopically expressing OPN-a (Figure 2G).

**Figure 3:**
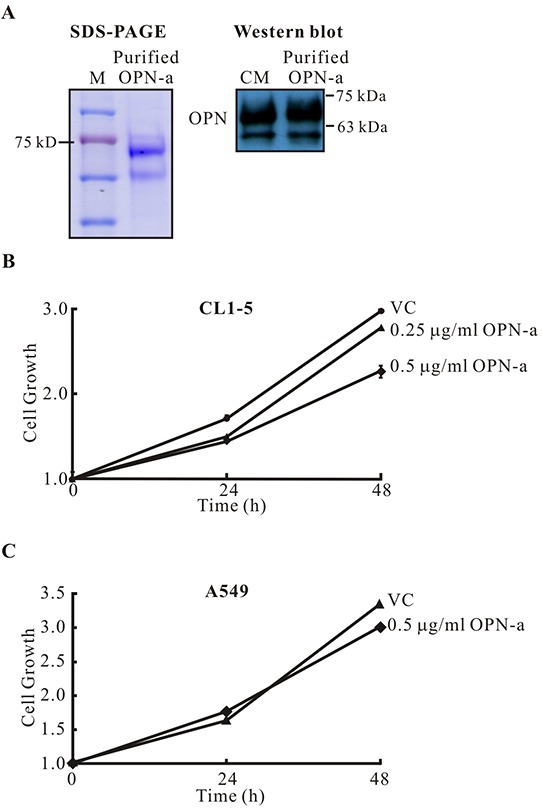
Purified OPN-a inhibits growth in CL1-5, but not A549, cells **A.** OPN-a-Myc-His fusion protein was purified with Ni-NTA agarose. Left panel, 10 μg of purified OPN-a were loaded. Right panel, Western blot analysis of purified OPN-a (3 μg) and CM/OPN-a (50 μL) detected with anti-OPN antibody (O17). **B.** Purified OPN-a reduced CL1-5 cancer cell growth. VC represents treatment of material from the control CL1-5/Vector stable clone using the same purification process. **C.** Purified OPN-a did not affect A549 cell growth.

To further confirm that OPN-a directly suppresses CL1-5 cell growth, we used Ni-NTA to purify OPN-a from CM (Figure 3A). As shown in Figure 3B and 3C, purified OPN-a efficiently suppressed CL1-5, but not A549, cell growth. Collectively, these results demonstrated that OPN-a, but not OPN-b or OPN-c, had the ability to inhibit cell growth. However, OPN-a did not suppress growth in all lung cancer cells.

### ITGβ3 is involved in OPN-a mediated growth inhibition

**Figure 4:**
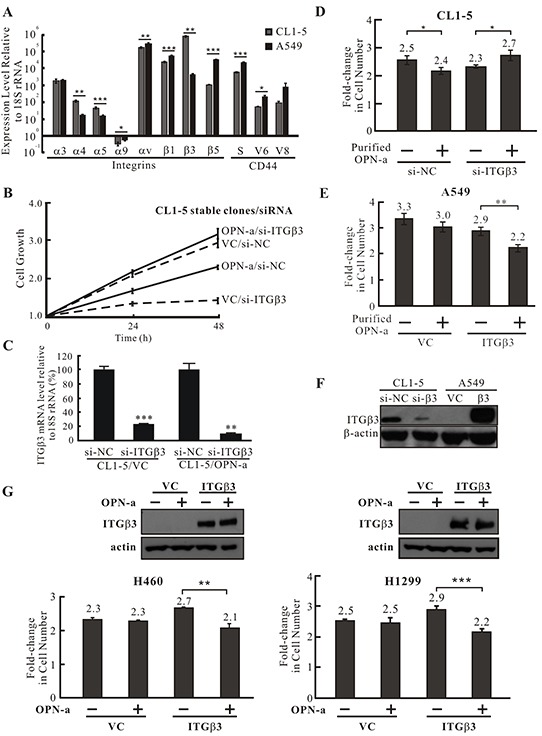
ITGβ3 is necessary for OPN-a-induced growth inhibition **A.** The expression of various integrin and CD44 isoform mRNAs was assessed by RT real-time PCR. Integrin α4, α5, and β3 expression levels were lower in A549 cells than in CL1-5 cells. The difference in ITGβ3 expression between CL1-5 and A549 cells was the most significant. *, *p* < 0.05; **, *p* < 0.01, ***, *p* < 0.001. **B.** Growth curves for OPN-a stably-expressing CL1-5 cells treated with si-ITGβ3 or si-NC. The OPN-a stable clone (OPN-a/si-NC) grew more slowly than VC/si-NC cells. ITGβ3 knockdown reversed OPN-a-induced growth inhibition in CL1-5 cells (OPN-a/si-ITGβ3). ITGβ3 also increased growth in CL1-5 cells (comparison between VC/si-NC and VC/si-ITGβ3). **C.** The efficacy of si-ITGβ3 in the CL1-5/OPN-a stable clone was measured by RT real-time PCR in cells in (B). **, *p* < 0.01, ***, *p* < 0.001. **D.** CL1-5 cells transfected with si-ITGβ3 or si-NC were treated with purified OPN-a. Similar to ITGβ3 knockdown in the CL1-5/OPN-a stable clone (B), ITGβ3 knockdown in CL1-5 cells reversed OPN-a-induced growth inhibition. *, *p* < 0.05. **E.** A549 cells exogenously expressing ITGβ3 were treated with purified OPN-a. Overexpression of ITGβ3 in greatly reduced A549 cell growth, suggesting that ITGβ3 is required for OPN-a-induced growth inhibition. **, *p* < 0.01. **F.** The expression of ITGβ3 protein in CL1-5/si-ITGβ3 and A549/ITGβ3 cells in (D & E). **G.** Purified OPN-a inhibited growth in H460/ITGβ3 and H1299/ITGβ3 cells, but did not affect growth in H460 and H1299 cells.

Secreted OPN can bind to various integrins and CD44 to activate signaling pathways. We thus performed real-time PCR to detect differences in the expression of integrins and CD44 in CL1-5 and A549 cells. As shown in Figure 4A, most detectable integrins and CD44 variants were more highly expressed in A549 cells; however, the expression of integrins α4, α5, and β3 was higher in CL1-5 cells. Among these three integrins, the disparity in β3 expression was greatest between the two cell types. To determine whether ITGβ3 plays a role in OPN-a-induced inhibition of CL1-5 cell growth, we reduced ITGβ3 expression in CL1-5/OPN-a and CL1-5/VC stable clones and monitored their growth rates. Knockdown of ITGβ3 expression enhanced growth in the CL1-5/OPN-a stable clone (Figure 4B, solid lines). Similarly, CL1-5 parental cells with reduced ITGβ3 expression that were treated with purified OPN-a grew faster than CL1-5 parental cells with normal ITGβ3 expression after the same treatment (Figure 4D). OPN-a also inhibited growth in A549 cells overexpressing ITGβ3 (Figure 4E). Furthermore, OPN-a-induced growth inhibition was also observed in H460 and H1299 cells overexpressing ITGβ3 (Figure 4G). These results demonstrated that ITGβ3 is necessary for OPN-a-induced growth inhibition. In the absence of OPN-a, ITGβ3 was essential for growth in CL1-5 cells (Figures 4B). The efficacy of si-ITGβ3 in CL1-5 cells and ectopic ITGβ3 expression are shown in Figure 4F.

### In the absence of ITGβ3, OPN-a induces cell growth via the CD44/NFkB pathway

**Figure 5:**
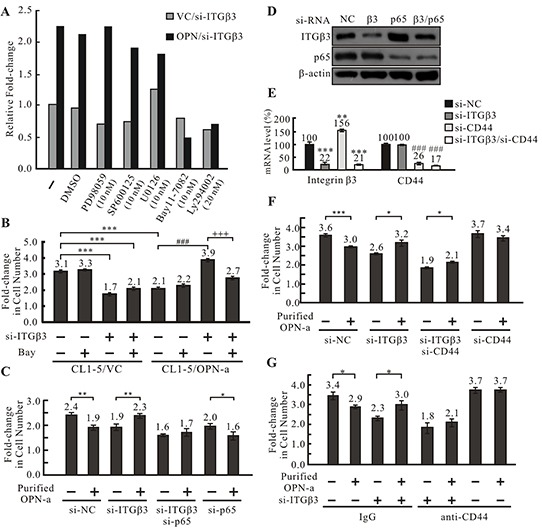
OPN-a induces cell growth following knockdown of ITGβ3 via the CD44/NFκB pathway **A.** Screening of pathways involved in OPN-a-induced growth inhibition. OPN-a stably expressing or empty vector CL1-5 cells were transiently transfected with si-ITGβ3 and treated with various pathway inhibitors. Fold-changes in cell numbers were determined by dividing cell numbers at 48 h by those at 0 h. Relative fold-change in cell number was calculated by dividing each fold-change in OPN/si-ITGβ3 cells by the fold-change in VC/si-ITGβ3 cells for each inhibitor treatment. NFκB inhibitor Bay11-7082 greatly inhibited growth in CL1-5/OPN-a cells with RNAi-induced ITGβ3 knockdown. PD98059: MAPK inhibitor; SP600125: JNK inhibitor; U0126: MEKK inhibitor; LY294002: PI3K inhibitor. **B.** The involvement of NFκB in OPN-a-induced growth inhibition. CL1-5/OPN-a and CL1-5/VC stable clones were transfected with si-ITGβ3 or si-NC and treated with/without 10 nM Bay11-7082 for 48 h. Ectopic expression of OPN-a reduced cell growth (lanes 1 & 5). ITGβ3 knockdown not only blocked OPN-a-induced growth inhibition, but also increased CL1-5/OPN-a cell growth (lanes 5 & 7). Bay11-7082 treatment abolished this increase in growth (lanes 7 & 8). Cell numbers were counted before (0 h) and after (48 h) treatment to calculate the fold-change in cell numbers. ***, *p* < 0.001; *^###^*, *p* < 0.001; *^+++^*, *p* < 0.001. **C.** The involvement of p65 in OPN-a-induced growth inhibition. CL1-5 cells were treated with si-ITGβ3, si-p65, or si-ITGβ3/si-p65 and incubated with/without 0.5 μg/mL purified OPN-a for 24 hr. Purified OPN-a inhibited growth in CL1-5/si-NC cells (lanes 1 & 2). Following ITGβ3 knockdown, OPN-a increased, rather than inhibited, cell growth (lanes 1 & 2 and lanes 3 & 4). siRNA-induced knockdown of both ITGβ3 and p65 eliminated this increase in growth (lanes 3 & 4 and 5 & 6), indicating that NFκB is required for OPN-a-induced increases in growth in CL1-5/si-ITGβ3 cells. The fold-change in cell number was calculated using cell numbers counted before and after OPN-a treatment *, *p* < 0.05; **, *p* < 0.01. **D.** The efficacy of si-ITGβ3 (5 pmol) and si-p65 (50 pmol) measured by Western blot analysis in the cells in (C). **E.** Efficiency of si-ITGβ3 (5 pmol) and si-CD44 (25 pmol) knockdown in CL1-5 cells in (F) measured by RT real time PCR. **, *p* < 0.01; ***, *p* < 0.001; *###*, *p* < 0.001. **F.** CD44 knockdown abolished OPN-a-induced increases in growth in CL1-5/si-ITGβ3 cells. Purified OPN-a inhibited growth in CL1-5 cells (lanes 1 & 2), and ITGβ3 knockdown enhanced cell growth (lanes 3 & 4). CD44 knockdown reduced this increase in cell growth (lanes 3 & 4 and lanes 5 & 6). The fold-change in cell number was calculated using cell numbers counted before and after OPN-a treatment. *, *p* < 0.05; **, *p* < 0.01; ***, *p* < 0.001. **G.** ITGβ3 knockdown in CL1-5 cells not only abolished OPN-a-induced growth inhibition, but also increased cell growth (lanes 1 & 2 and lanes 3 & 4). Blocking CD44 with 0.5 μg of CD44 antibody reduced the OPN-a-induced increase in growth in CL1-5/si-ITGβ3 cells (lanes 3 & 4 and 5 & 6). IgG is the negative control for the CD44 antibody. *, *p* < 0.05.

In the absence of ITGβ3, OPN-a enhanced CL1-5 cell growth (Figure 4B & 4D). Since OPN binds to various integrins and CD44, it is conceivable that OPN-a could bind to another receptor to enhance growth in the absence of ITGβ3. We thus treated CL1-5/OPN-a and CL1-5/VC stable clones transfected with si-ITGβ3 with various signaling pathway inhibitors to screen for pathways involved in OPN-a-induced growth in the absence of ITGβ3. Inhibition of NFκB activity with Bay-11-7082 blocked OPN-a-induced growth (Figure 5A). To confirm this result, we treated CL1-5/OPN-a and CL1-5/VC stable clones transiently transfected with si-ITGβ3 or si-NC with Bay-11-7082 and measured cell growth. As shown in Figure 5B, ITGβ3 knockdown in the CL1-5/OPN-a stable clone enhanced cell growth (lanes 5 & 7). However, treatment with 10 nM Bay-11-7082 inhibited OPN-a-induced growth in CL1-5/si-ITGβ3 cells (Figure 5B, lanes 7 & 8), but did not inhibit growth in CL1-5/VC cells regardless of ITGβ3 expression (Figure 5B, lanes 1 & 2; lanes 3 & 4). Knockdown of both ITGβ3 and NFκB subunit p65 blocked OPN-a-induced growth in CL1-5 cells (Figure 5C, lanes 5 & 6) compared to CL1-5 cells with ITGβ3 knockdown alone (lanes 3 & 4). The efficacies of si-ITGβ3 and si-p65 are shown in Figure 5D.

NFκB is the down-stream effector of CD44 [26]. To determine whether OPN-a promotes growth by binding to CD44 in the absence of ITGβ3, we blocked CD44 expression in CL1-5/si-ITGβ3 cells using RNAi (Figure 5E) and examined the effect of OPN-a on growth. ITGβ3 and CD44 double knockdown inhibited OPN-a-induced cell growth (Figure 5F, lanes 5 & 6) compared to cells with ITGβ3 knockdown alone (Figure 5F, lanes 3 & 4), suggesting that, when ITGβ3 expression is reduced, OPN-a instead binds to the CD44 receptor and enhances cell growth via the NFκB signaling pathway. This OPN-a-induced growth did not occur following knockdown of both CD44 and ITGβ3. To confirm these observations, the effect of OPN-a on CL1-5/si-ITGβ3 cell growth when an anti-CD44 antibody was used to block CD44 function was determined. Anti-CD44 reduced OPN-a-induced growth in the absence of integrin β3 (Figure 5G, lanes 3 & 4 and 5 & 6).

**Figure 6:**
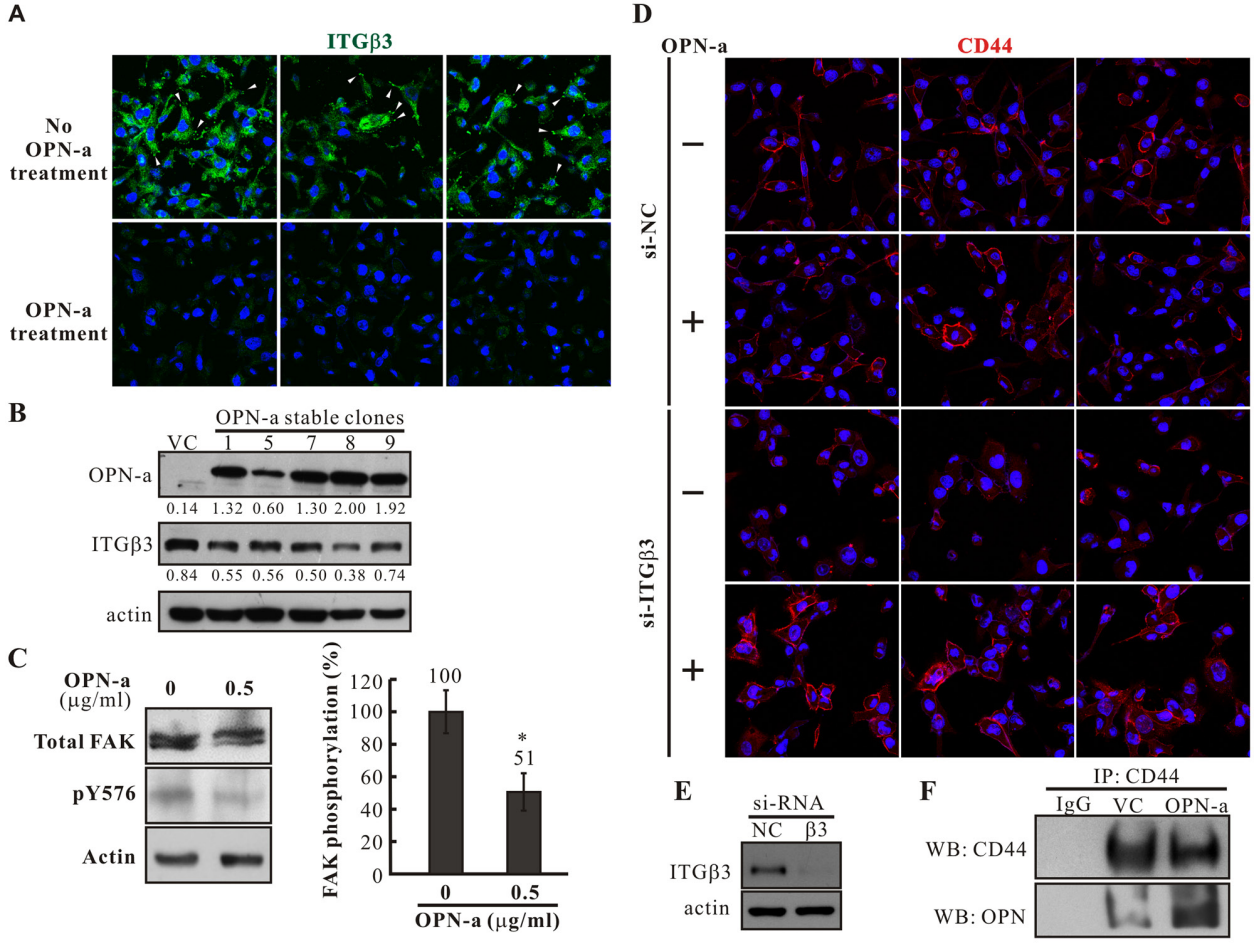
The effects of OPN-a on ITGβ3 and CD44 membrane distribution in CL1-5 cells **A.** CL1-5 cells were treated with/without OPN-a and immunostained with anti-ITGβ3 antibody. ITGβ3 staining appeared as punctuated signals (arrow heads) in the basal section of confocal images in untreated cells; OPN-a treatment greatly decreased both punctuated and overall signals. **B.** Expression of ITGβ3 was reduced in OPN-a stably transfected cells in Western blot analysis. **C.** FAK Y576 phosphorylation was decreased in CL1-5 cells treated with 0.5 μg/mL of purified OPN-a compared to untreated cells. *, *p* < 0.05. **D.** CL1-5 cells treated with/without si-ITGβ3 were incubated with/without purified OPN-a. Cells were then immunostained with anti-CD44. When ITGβ3 expression was reduced, purified OPN-a increased membrane-localized CD44 levels. **E.** The efficacy of si-ITGβ3 in cells in (D). **F.** Increased co-immunoprecipitation of OPN-a with CD44 was observed in the CL1-5/OPN-a stable clone compared to the CL1-5/VC clone.

To investigate the effect of OPN-a on surface ITGβ3 expression, untreated CL1-5 cells and cells treated with 0.5 μg/mL purified OPN-a were immunostained with anti-ITGβ3. ITGβ3 staining appeared as punctuated fluorescence signals in the basal sections of confocal images of untreated CL1-5 cells. However, OPN-a treatment greatly reduced the punctuated and overall ITGβ3 signals (Figure 6A). Consistent with this finding, CL1-5 cells overexpressing OPN-a had lower ITGβ3 levels (Figure 6B). Furthermore, OPN-a treatment reduced the phosphorylation of FAK, the downstream effector of integrin, suggesting that OPN-a treatment attenuated ITGβ3 activity (Figure 6C). When ITGβ3 was present, OPN-a did not affect the membrane distribution of CD44. However, following knockdown of ITGβ3 expression via si-RNA, OPN-a treatment increased membrane CD44 levels (Figure 6D). Indeed, co-immunoprecipitation of OPN-a with CD44 was increased in the CL1-5/OPN-a stable clone compared to the CL1-5/VC clone (Figure 6F). These results suggest that binding of OPN-a to ITGβ3 decreases ITGβ3 levels at focus adhesion points or focal adhesion formation. When ITGβ3 levels were reduced, OPN-a bound to, stabilized, and activated the membrane-localized alternative receptor CD44. Thus, CD44, ITGβ3, and OPN-a interact with each other to affect CL1-5 cell growth.

## DISCUSSION

The results of this study showed that OPN-a can either increase or inhibit growth in cancer cells depending on the expression of its receptors. Levels of ITGβ3 and CD44 specifically determine the effects of OPN-a on growth in lung cancer cells. CL1-5 cells express high levels of ITGβ3, while A549 cells express low levels of ITGβ3. Both of these cell types express CD44, but CD44 levels are higher in A549 cells. OPN-a treatment reduced growth in CL1-5, A549/ITGβ3, H460/ITGβ3, and H1299/ITGβ3 cells compared to untreated cells. These results suggest that ITGβ3 is necessary for OPN-a-induced growth inhibition. Immunostaining revealed that OPN-a treatment greatly diminished membrane and overall ITGβ3 levels. It is possible that the binding of OPN-a to ITGβ3 causes internalization and destabilization of ITGβ3 as well as other associated growth receptors.

**Figure 7:**
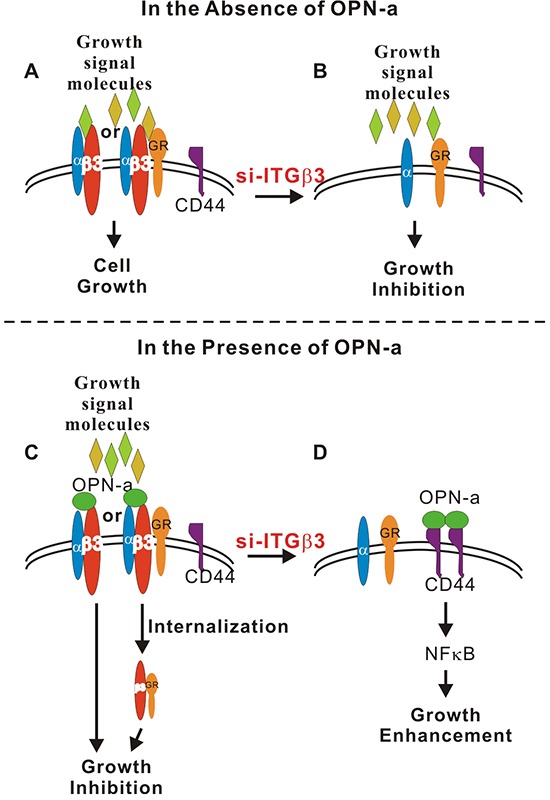
Schematic diagram of OPN-a- and ITGβ3-mediated growth regulation **A.** In the absence of OPN-a, ITGβ3 binds to a signaling molecule (growth signal, environmental molecule or growth receptor) to maintain growth in CL1-5 cells. **B.** Knockdown of ITGβ3 reduces cell growth. **C.** OPN-a might compete with growth signal molecules to bind ITGβ3 or cause internalization of ITGβ3 or the ITGβ3/growth receptor complex, thus reducing cell growth. **D.** Knockdown of ITGβ3 enables OPN-a to bind to the alternate receptor CD44, which increases CD44 membrane localization and increases growth by activating NFκB.

Knockdown of ITGβ3 expression in CL1-5 cells not only abolished OPN-a-induced growth inhibition, but also enhanced OPN-a-induced cell growth. In the absence of ITGβ3, OPN-a increased cell growth via the CD44/NFκB pathway. In line with this result, OPN-a treatment greatly enhanced membrane CD44 levels following si-RNA-induced ITGβ3 knockdown. Based on these results, we propose that, in the absence of OPN-a, ITGβ3 increases growth by binding to a growth factor or activating a growth receptor (Figure 7A). Increased OPN-a levels in the surrounding environment block the binding of ITGβ3 to growth factors, thus reducing cell growth. Alternatively, binding of OPN-a to ITGβ3 might result in the internalization of ITGβ3 as well as associated growth receptors, thus inhibiting cell growth. (Figure 7C). In contrast, when ITGβ3 levels are low, OPN-a binds to, stabilizes, and activates CD44, which in turn activates growth signals (Figure 7D). Finally, in the absence of OPN-a, ITGβ3 is essential for CL1-5 cell growth (Figure 7B).

OPN mediates cell migration through various integrins, including αvβ1, αvβ3, αvβ5, α4β1, α5β1, α8β1, and α9β1 (reviewed in [13]). Among integrin heterodimers, αvβ3 is most commonly associated with OPN-induced malignancy. The αvβ3 complex is involved in OPN-induced migration and invasion in tumor cells [27, 28], as well as OPN-induced endothelial cell migration and angiogenesis [29, 30]. In addition, ligation of OPN to CD44 increases cell adhesion, invasion, and transformation [13, 31]. OPN directly interacts with integrin β3 and CD44 [32, 33]; the RGD and SVVYGLR sequences in OPN are responsible for its interaction with integrin [34], while CD44 binding sites in the OPN sequence have not yet been identified. Interestingly, OPN acts as a bridge molecule between CD44 variants and integrins to stimulate motility in murine cells [5]. The interaction between OPN and αvβ3 increases CD44v6 expression in HepG2 cells and enhances cell adhesion to hyaluronic acid [35]. Additionally, binding of OPN-a to CD44 variants activates integrins through Src, thus promoting ECM-derived survival signals in gastric cancer [36].

Although up-regulation of OPN in plasma or cancer tissues is associated with poor cancer prognosis, our results clearly demonstrate that OPN-a, but not OPN-b or -c, decreased CL1-5 cell growth. The reason for which OPN-a overexpression suppresses growth in these cancer cells is unknown. OPN promotes the migration of immune cells toward a wound site. However, tumor-derived OPN has been shown to inhibit macrophage function and promote tumor growth [37]. The ability of tumor cells to evade host immunity may result from decreased NO production by macrophages in response to tumor-derived OPN [38]. The effects of different OPN isoforms on immunity are also unknown. The OPN-a splicing variant, which was the most highly expressed in tumor cells, may help to inhibit immune surveillance. In addition, we found that OPN-a treated fibroblasts may secrete molecules that enhance lung cancer cell growth. (data not shown)

Our study revealed that ITGβ3 and CD44 expression levels determine whether OPN-a inhibits or enhances growth in lung cancer cells. Furthermore, the effect of ITGβ3 on cancer cell growth is determined by environmental levels of OPN. OPN and ITGβ3 are attractive targets for cancer therapy, and ITGβ3 target therapy (such as cyclic RGD) has been widely investigated. Our study suggests that ITGβ3 target therapy would effectively inhibit the growth of OPN-negative cells, but may also increase the growth of OPN-positive cells.

## MATERIALS AND METHODS

### Cell culture and treatment

The NCI-A549, NCI-H23, NCI-H441, and NCI-H1355 lung adenocarcinoma (AD) cell lines, NCI-H358 and NCI-H1299 non-small cell lung cancer (NSCLC), and NCI-H460, NCI-H661 large cell lung carcinoma cell lines were obtained from the Bioresource Collection and Research Center (BCRC, Taiwan). CL1-0, CL1-3, and CL1-5 lung AD cell lines were kindly provided by Dr. P-C Yang (Department of Internal Medicine, National Taiwan University, Taipei, Taiwan). For cell growth assay, A549 and H1299 cells were maintained in DMEM medium containing 10% fetal bovine serum (FBS). CL1-0, CL1-5, and H460 cells were maintained in RPMI1640 containing 10% FBS. RPMI, DMEM and FBS were purchased from Gibco.

### Cloning and transfection

*OPN-a* and *-b* were PCR amplified from CL1-0 cDNA and cloned into pcDNA™ 3.1/*myc*-His(−)A vectors. To obtain OPN-c, PCR-based site-directed mutagenesis was carried out to delete exon 4 in the OPN-a construct. ITGβ3 was PCR amplified (forward primer: 5′-GCGACGGTCCGGCCACCATGCGAGCGCGG-3′; reverse primer: 5′-GCCACGGACCGCAGTGCCCCTGTACGTGAT-3′) from CL1-5 cDNA and ligated with a T/A vector. After sequencing, ITGβ3 was subcloned into the RsrII site in pcDNA 3.1/myc-His(−)A. The pcDNA vector was engineered by inserting an adaptor with a RsrII restriction site (F’ oligo: 5′-GATCCGGTCCGCAC-3′; R’ oligo: 5′-GATCGTGCGGACCG-3′) into the BamHI site and was mutated at the RsrII site in the Neo^r^ gene. Transfection was performed with Lipofectamine™ 2000 (Invitrogen) transfection reagent according to the manufacturer's instructions. The transfected CL1-5 cells were treated with G418 (500 μg/mL) (Sigma-Aldrich) for 3–4 weeks to select stable clones. For RNA interference, siRNA (si-*ITGβ3*: 5′-GGACUACGCUCUCUGAAUUTT-3′, si-CD44: 5′-UAUUCCACGUGGAGAAAAATT-3′, si-p65: 5′-CCUUUCUCAUCCCAUCUUUTT-3′, or si-NC: 5′-UUCUCCGAACGUGUCACGUTT-3′ (provided by MDBio, Inc, Taiwan)) was transfected into cell lines using Lipofectamine, and cell lines were then cultured for 24 h. The transfected cells were then transferred into appropriate media for analyses.

### Western blot analysis

Cells were lysed with lysis buffer containing 0.5% NP-40, 50 mM Tris-Cl (pH 7.5), 1 mM EDTA, and protease inhibitor cocktail (Roche) for 3 min. Then, cell debris was removed by centrifugation and the protein concentration was determined using the Bradford protein assay kit (Bio-Rad). Equal amounts of protein were separated on sodium dodecyl sulfate-polyacrylamide (SDS-PAGE) gels and then transferred to polyvinylidene fluoride (PVDF) membranes (Perkin Elmer). After blocking, the membranes were incubated with anti-Flag (Sigma), anti-OPN (#10A16 or #O17, IBL10011, 18625.), anti-ITGβ3 (Abcam ab75872), or anti-p65 antibodies (Millipore, 06-418) at 4°C overnight, followed by incubation with horseradish peroxidase-conjugated secondary antibody at room temperature for 1 hour. The blots were visualized using an enhanced chemiluminescence (ECL) kit (Perkin Elmer).

### Immunofluorescence staining

CL1-5 cells (3×10^5^ cells/well) were seeded onto 6-well plates and cultured to 70% confluence. Cells were then transfected with or without si-NC or si-ITGβ3 using Lipofectamine and incubated for 24 h. The transfected cells were seeded (1.5×10^5^ cells) onto cover slips. After 16 h incubation, cells were treated with 0.5 μg/mL OPN-a for 24 h. The cells were then fixed with 100% methanol for 10 min at −20°C, followed by blocking with 3% BSA for another 60 min at room temperature. The fixed cells were then probed with anti-integrin β3 (1:200 dilution; Abcam ab75872) or CD44 (1:200 dilution; GeneTex Gtx102111) antibody for 1 h at room temperature, followed by incubation with a TRITC or F488-conjugated goat anti-rabbit IgG antibody (1:500 dilution; Sigma, St. Louis, MO, USA) for 1 h at room temperature. The cells were then washed with PBS three times, stained with DAPI for 15 min, and mounted with mounting solution. The expression and location of target proteins were observed with a laser scanning confocal microscope.

### Immunoprecipitation

For immunoprecipitation of CD44, 5×10^6^ CL1-5/OPN-a cells were lysed with RIPA buffer (50 mM Tris, pH 7.4, 150 mM NaCl, 1% NP-40, 1% sodium deoxycholate, 2 mM EDTA 2, 1 mM PMSF, protease inhibitor, and phosphatase inhibitor). The lysate (1 mg protein) was incubated with 4 μg anti-CD44 (Abcam, ab157107) antibody overnight at 4°C. Washed protein G beads (50 μL, MagQu, MF-PRG-3000) were then added to the protein-antibody mixture followed by incubation for 5 h at 4°C. After washing, the protein complex was recovered by heating at 95°C for 3 min in loading buffer. CD44 and OPN were resolved with SDS-PAGE and immunoblots were subsequently probed with anti-CD44 (Abcam ab157107) and anti-OPN (O17, IBL 18625) antibodies.

### Reverse transcription and real-time PCR

Total RNA (2 μg) was extracted from tissues and cell lines with TRIZOL Reagent (Invitrogen), treated with RQ1 DNase (Promega #M6101), and subjected to cDNA synthesis according to the manufacturer's instructions (Epicentre RT80125K). All real-time PCR reactions were carried out with the ABI StepOne system using FastStart Universal SYBR Green Master (ROX) (Roche). The following primers were used for real-time PCR: *18S rRNA*-F, 5′-TCGGAACTGAGGCCATGA-3′ and *18S rRNA*-R, 5′-CCGGTCGGCATCGTTTA-3′. Primers for detection of OPN isoforms were as follows: 5′-CATCACCTGTGCCATACCA-3′ (located at exon 2) and 5′-GGCTGTCCCAATCAGA-3′ (located at exon 6). Primers for detection of integrins were as follows: *ITGα3*-F, 5′- TGCTCACCCCTCACTCCT-3′; *ITGα3*-R, 5′- GGCAGTCCCAGCTTCTCT-3′; *ITGα4*-F, 5′- GCT CGGGAGCAGTAATGAA-3′; *ITGα4*-R, 5′- ATCTGCA CGGCCATTGTA-3′; *ITGα5*-F, 5′- CCCTGCCGCTCAG ATTT-3′; *ITGα5*-R, 5′- CTGGCCTGGCGAGTCT-3′; *ITGαv*-F, 5′- CAAAGCAAACACCACCCA-3′; *ITGα9*-F, 5′- GTGATGCCGGTGGGATA-3′; *ITGα9*-R, 5′- CCGGGAGGAAGATGGA-3′; *ITGαv*-R, 5′-GGG GCACAGGCCAA-3′; *ITGβ1*-F, 5′-CGTAGCAAAG GAACAGCAGA-3′; *ITGβ1*-R, 5′- AGTCCGAAGTA ATCCTCCTCA-3′; *ITGβ3*-F, 5′- GGCCCCTCA GCGACA-3′; *ITGβ3*-R, 5′- AATGCCCCGAAGCCA-3′; *ITGβ5*-F, 5′- TGGCTGGCGAAAGGAT-3′; *ITGβ5*-R, 5′- GCAAGGCAAGGGATGGAT-3′. Primers for detection of CD44 variants were as follows: *CD44*-F, 5′- TCTACCCCAGCAACCCTACT-3′; *CD44*-R, 5′- CCACCTTCTTGACTCCCATGT-3′; *CD44v6*-F, 5′- TCC AGGCAACTCCTAGTAGTACA-3′; *CD44v6*-R, 5′- GGT GTGAGATTGGGTTGAA-3′; *CD44v8*-F, 5′- CGC TTCAGCCTACTGCAA-3′; *CD44v8*-R, 5′- GGGT CTCTTCTTCCACCTGT-3′.

### Cell growth assay

Cells were cultured to 70% confluence. After detachment, 3 × 10^4^ cells were seeded onto 12-well plates and cultured in RPMI-1640 (for CL1-5) or DMEM (for A549) containing 10% FBS with or without CM or purified OPN-a. After 16 h of incubation, cells were harvested and viable cells were counted; this time point was designated as 0 h. After an additional 24 h of incubation, cells were harvested and viable cells were counted; this time point was designated as 24 h. For pathway inhibitor screening, 3 × 10^4^ cells were inoculated and incubated for 16 h. Cells were then either harvested before treatment (designated as 0 hr) or treated with various inhibitors for 48 h (designated 48 h), and viable cells were counted. Fold-change in cell number was calculated by dividing viable cell numbers at 24 h (or 48 h) by those at 0 h. Relative fold-change in cell number was calculated by dividing each fold-change in treatment cells by the fold-change in control cells.

### Focus formation assay

For the focus formation assay, 200 cells were seeded onto a 3.5 cm dish. Following incubation for 7–9 days, colonies were fixed with 100% ice-cold methanol for 30 min, followed by staining with 20% Giemsa for 30 min.

### Purification of OPN-a from CM/OPN-a

To purify ectopically expressed OPN-a, the CL1-5/OPN-a-Myc-His stable clone or CL1-5/VC cells were grown to 100% confluence in 10 cm dishes. The medium was replaced with 4 mL RPMI serum-free medium, followed by incubation at 37°C for 24 h. About 120 mL of CM were collected and concentrated with a Vivaspin® 15R Centrifugal Concentrator to about 5 mL, followed by incubation with an equal volume of Ni-NTA agarose beads (QIAGEN) at 4°C for 2 h. After washing with wash buffer (50 mM Na_2_HPO_4_, 300 mM NaCl, 20 mM imidazole, 0.05% Tween 20, pH 8.0), OPN-a was eluted with elution buffer (50 mM Na_2_HPO_4_, 300 mM NaCl, 250 mM imidazole, 0.05% Tween 20, pH 8.0). To eliminate imidazole, the eluent was dialyzed against 1xPBS. The purified OPN-a concentration was determined using a Bradford protein assay kit (Bio-Rad).

### Preparation of conditioned medium

Conditioned medium (CM) was prepared from cells transiently or stably transfected with different OPN splicing variants. All cells were grown to full confluence in growth medium with 10% FBS, which was then replaced with 2 mL of fresh medium followed by incubation at 37°C for 24 h in 3.5 cm dishes. After removing cells debris by centrifugation, CM was mixed with equal volumes of fresh media containing 10% FBS for cell culture assays.

### Statistical analysis

All growth assays, except for the pathway inhibitor analysis experiment, were repeated independently at least times with three replicates in each experiment. Values are presented as mean ± SD. GraphPad Prism 5.0 software (GraphPad Software, Inc., San Diego, CA, http://www.graphpad.com) was used for statistical analyses. Statistical significance was assessed by Student's *t*-tests and is indicated as follows: *, *p* < 0.05; **, p < 0.01; ***, p < 0.001.; *^###^, p* < 0.001; *^+++^, p* <0.001.
